# The interplay of sleep architecture and exercise in executive function of middle-aged and older adults

**DOI:** 10.3389/fneur.2026.1839841

**Published:** 2026-05-21

**Authors:** WenHui Zheng, LiYing Huang, Mian Wu, YuHe Chen

**Affiliations:** 1 School of Sports Training, Guangzhou Sport University, Guangzhou, China; 2 School of Sports and Health Sciences, Guangzhou Sport University, Guangzhou, China; 3 Guangdong Provincial Key Laboratory of Human Movement Science, Guangzhou Sport University, Guangzhou, China; 4 School of Physical Education and Sports Science, South China Normal University, Guangzhou, China; 5 School of Sports Science, Zhuhai University of Science and Technology, Zhuhai, China

**Keywords:** brain network dynamics, executive function, neuromodulatory systems, physical exercise, sleep architecture

## Abstract

Executive function decline in middle-aged and older adults is a significant public health concern, with sleep disturbances and physical inactivity being two major modifiable risk factors. Existing evidence suggests bidirectional associations between sleep and exercise, with both factors potentially influencing cognitive function. This review synthesizes current evidence on the interplay among exercise, sleep architecture, and executive function in aging populations. We first discuss the noradrenergic and adenosinergic systems as shared neuromodulatory substrates underlying the reciprocal regulation of sleep and exercise. We then review evidence linking slow-wave sleep (SWS) to exercise-induced neuroplasticity and sleep spindles to memory consolidation. The glymphatic system is presented as a sleep-dominant clearance mechanism that may interact with exercise. At the brain network level, we summarize how sleep and exercise are, respectively, associated with the dynamic balance between the central executive network and the default mode network. Furthermore, subcomponent-specific associations are examined: SWS duration correlates with inhibitory control and working memory, whereas REM sleep is linked to cognitive flexibility, and resistance or mind–body exercises show selective benefits for distinct executive domains. Nonlinear dose-timing effects are also considered, such as the optimal moderate-intensity aerobic exercise for preserving SWS and the morning exercise preference for aligning with circadian rhythms in older adults. Collectively, this review provides a theoretical basis for understanding how physical activity and sleep architecture jointly influence executive function in middle-aged and older adults. It highlights convergent physiological pathways—ranging from molecular neuromodulators and glymphatic clearance to large-scale brain network dynamics—that may guide future mechanistic studies and intervention strategies for age-related cognitive decline.

## 1 Introduction

In 2025, the global population aged 60 and above reached approximately 1.2 billion, accounting for 12.9% of the total population, with projections indicating a rise to 2.1 billion by 2050 (1, 2). With the acceleration of global population aging, cognitive decline has emerged as a pressing public health issue (3). Executive function, consisting of inhibitory control, working memory and cognitive flexibility, served as the core component of cognitive ability and was critical for maintaining independent living in older adults (4). Executive function declined markedly after the age of 45, and its neural basis relied on a distributed network encompassing the prefrontal cortex, anterior cingulate cortex and parietal lobe (5). Regular physical activity and sufficient high-quality sleep were two pivotal modifiable factors that regulated cognitive function and delayed cognitive deterioration in middle-aged and older populations (6). Physical activity improved cognitive outcomes by modulating neural, endocrine and immune systems (7), while sleep exerted important roles in sustaining neurotransmitter homeostasis, clearing metabolic waste and maintaining neuroplasticity (8).

Regular exercise yielded beneficial effects on executive function among middle-aged and older adults (8). Accumulated studies demonstrated that aerobic exercise, resistance training, as well as mind–body exercises such as Tai Chi, ameliorated subcomponents of executive function, including inhibitory control, working memory and cognitive flexibility (7, 9). Such beneficial effects were mediated by multiple mechanisms, including enhanced prefrontal cortical plasticity, elevated release of neurotrophic factors and optimized cerebral network connectivity (9, 10).

Sleep architecture referred to the temporal organization and periodic alterations of distinct sleep stages, mainly involving slow-wave sleep (SWS) and sleep spindles during non-rapid eye movement (NREM) sleep, as well as rapid eye movement (REM) sleep (10). SWS predominantly occurred in the first half of the night and was closely linked to synaptic homeostasis regulation, metabolic waste elimination and long-term memory consolidation. Sleep spindles manifested during stage 2 NREM sleep, modulated thalamocortical rhythmic oscillations, facilitated hippocampal-neocortical crosstalk, and played a vital role in information reactivation and memory storage (11). In middle-aged and older individuals, the duration of SWS and spindle density decreased significantly with aging, and these age-related alterations were correlated with the deterioration of working memory updating, inhibitory control and cognitive flexibility (12).

Although previous studies provided substantial evidence separately linking sleep, exercise, and executive function, an integrated mechanistic understanding of how these factors interact remains lacking. The present review synthesized current evidence regarding the bidirectional regulation between sleep and exercise as well as their combined impacts on executive function in middle-aged and older adults. It specifically explored how the interactions between sleep architecture and physical activity modulated executive function, with a focus on shared neuromodulatory systems, reciprocal relationships between sleep structure and exercise, and large-scale brain network dynamics across molecular and systemic levels. This review aimed to construct a conceptual framework for illustrating the joint effects of physical activity and sleep architecture on executive function in aging populations and provided theoretical references for future precise cognitive intervention.

## 2 Physiological mechanisms underlying the interaction between sleep architecture and physical activity

### 2.1 Effects of physical activity on slow-wave sleep and sleep spindles

Aging was characterized by pronounced reductions in SWS duration and sleep spindle density among middle-aged and older adults (12). Previous studies found that SWS increased following physical activity, especially after the acquisition of new motor skills, and the enhancement of local slow-wave activity was positively correlated with motor learning outcomes (13). During SWS, synchronized neuronal oscillations facilitated synaptic pruning and memory consolidation, accompanied by elevated secretion of brain-derived neurotrophic factor (BDNF) (14). Functional neuroimaging evidence indicated that long-term exercise intervention increased gray matter volume in the hippocampus and prefrontal cortex, and such structural plasticity was closely associated with the integrity of SWS (15).

Sleep spindles were 11–16 Hz oscillations that occurred during NREM sleep and originated from thalamocortical circuits, which were tightly coupled with memory consolidation (16). Researchers observed that sleep spindle density elevated after motor skill learning, and the increased spindle density positively correlated with improvements in motor performance on the subsequent day (17). Advanced analyses revealed that spindles periodically appeared in clustered bursts, and this temporal pattern provided a critical time window for the repeated reactivation of memory traces (18).

SWS and sleep spindles exhibited functional coupling. The coordination between slow oscillations and spindles during NREM sleep promoted hippocampal-neocortical communication, which was essential for long-term memory consolidation (19). Several studies have suggested that physical activity strengthened such coordination (20), thereby facilitating synaptic plasticity, memory consolidation and neural network stability—the core physiological processes underlying executive function improvement (21).

### 2.2 Shared neuromodulatory systems: the link between norepinephrine and adenosine

The noradrenergic system exerted a core regulatory role in sleep architecture. The locus coeruleus (LC) acted as the primary source of central norepinephrine and remained nearly quiescent during SWS. This low-activity state sustained SWS maintenance and promoted glymphatic clearance (22). Physical activity transiently increased norepinephrine levels; however, evening exercise induced persistent sympathetic activation, which delayed sleep onset and reduced SWS duration (23). Such modulatory effects on SWS indirectly affected cognitive flexibility and inhibitory control in older adults (24).

The adenosine system mediated the accumulation of sleep pressure (Process S). Adenosine concentrations gradually increased during wakefulness, and moderate-intensity aerobic exercise accelerated this accumulative process (25). Adenosine was cleared during sleep, and its circadian rhythm presented temporal coupling with the diurnal distribution of SWS (26). By regulating adenosine signaling, physical activity altered the release rhythm of sleep pressure and further modulated SWS duration and spindle density (27).

### 2.3 Sleep-dependent glymphatic clearance mechanism

The glymphatic system represented a significant discovery in neuroscience (28). Animal studies have confirmed that cerebrospinal fluid (CSF) infiltrated the brain parenchyma along perivascular spaces during sleep, which accelerated the clearance of metabolic waste such as *β*-amyloid and tau proteins (29). This process was most active during SWS and depended on normal expression of aquaporin-4 (AQP4) (30).

Physical activity might regulate glymphatic function through multiple pathways, although much of the evidence comes from animal studies. First, exercise elevated cardiac output and cerebral blood flow, which indirectly enhanced CSF circulation (31). Second, exercise-induced lactate signaling modulated the expression of AQP4 (32). Third, exercise optimized sleep architecture, particularly by prolonging SWS duration, and thus extended the functional time window for glymphatic clearance (33). Nevertheless, relevant evidence was predominantly derived from animal experiments, and clinical studies focusing on older adults with age-related glymphatic dysfunction remained insufficient (34).

### 2.4 Large-scale brain network dynamics

Executive function depends on the coordinated activity of large-scale brain networks, and aging disrupts such neural coordination in middle-aged and older individuals (35). The triple-network model identified three core networks: the central executive network (CEN), the default mode network (DMN), and the salience network (SN) (36). Under normal cognitive states, CEN and SN were synergistically activated during tasks, while DMN was suppressed, forming a dynamic balance (37). This homeostatic balance was disrupted in the aging brain, manifested as weakened task-induced DMN suppression, reduced CEN activation and impaired salience detection of the SN (38). Such network imbalance was recognized as the neural correlate of age-related executive function decline (39).

In addition to sleep architecture disturbances, total sleep deprivation and physical activity exerted opposing regulatory effects on brain network dynamics. Acute sleep deprivation reduced activation in the dorsolateral prefrontal cortex, induced overactivation of the posterior cingulate cortex, and weakened the anti-coupling between the CEN and DMN (40, 41). In contrast, a single session of aerobic exercise strengthened CEN-SN coupling, inhibited excessive DMN activity, and improved the activation efficiency of task-related brain regions (42). Longitudinal studies further validated that regular exercise increased intra-CEN connectivity and enhanced negative CEN-DMN coupling (43).

The three core subcomponents of executive function, namely inhibitory control, working memory, and cognitive flexibility, relied on partially overlapping yet dissociable neural circuits (44). Inhibitory control was modulated by the right inferior frontal gyrus–supplementary motor area network and GABAergic transmission (24), and SWS duration was positively associated with inhibitory control performance, with resistance training exerting selective beneficial effects on this domain (45, 46). Working memory depended on hippocampal-prefrontal synergy (47). Aerobic exercise showed the most robust evidence for working memory improvement via BDNF-mediated hippocampal neurogenesis, and sleep within 3–4 h after exercise was critical for memory consolidation (48, 49). Cognitive flexibility was regulated by the prefrontal-striatal network and dopaminergic modulation (50). REM sleep facilitated the improvement of cognitive flexibility, and mind–body exercises including Tai Chi exerted prominent regulatory effects on this cognitive subdomain (51, 52).

**Figure 1:**
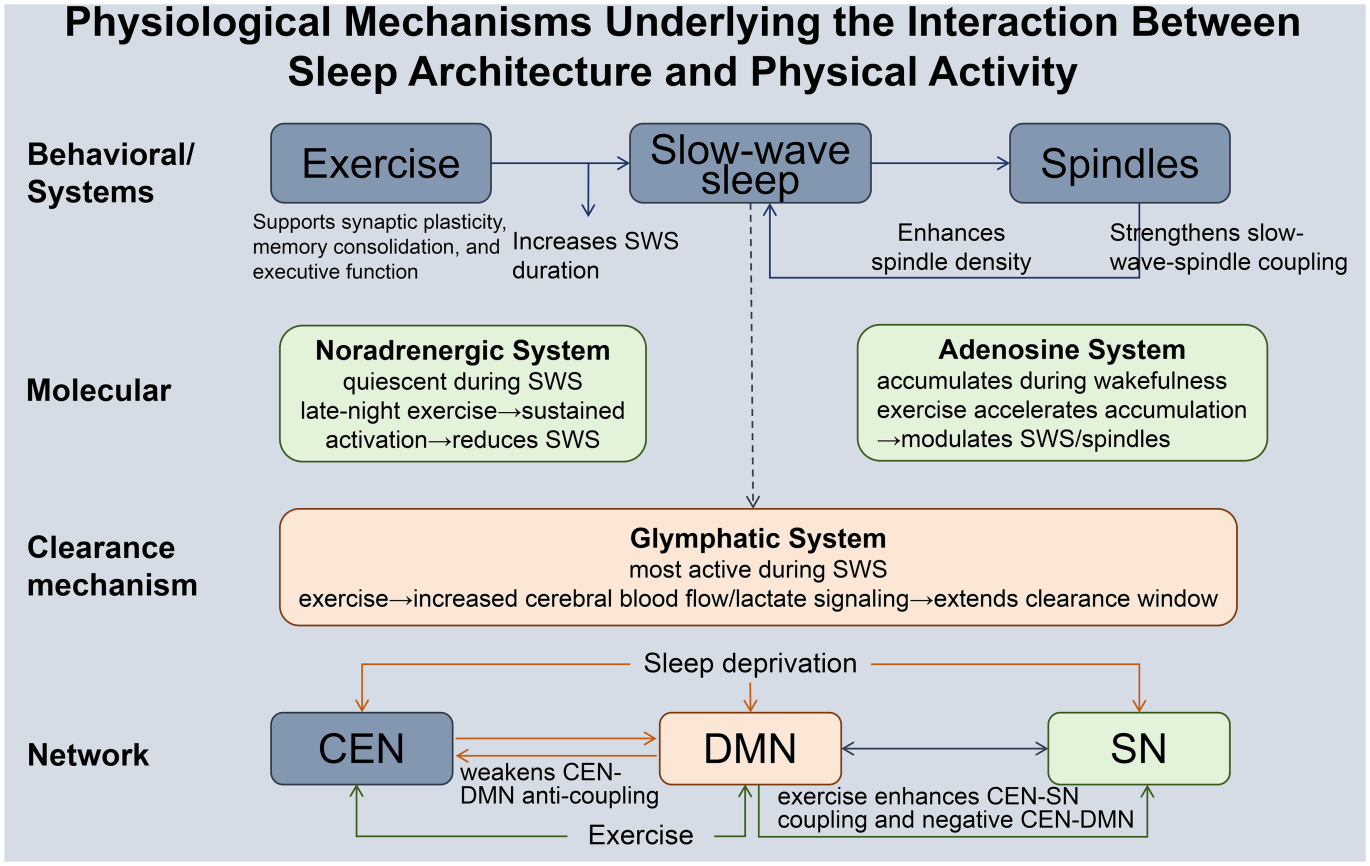
Physiological mechanisms underlying the interaction between sleep architecture and physical activity. SWS, Slow-Wave Sleep; CEN, Central Executive Network; DMN, Default Mode Network; SN, Salience Network.

Figure 1 provides a schematic summary of the multilevel physiological mechanisms through which sleep architecture and physical activity interact to influence executive function in middle-aged and older adults.

## 3 Implications for intervention

### 3.1 Exercise dose and sleep architecture

Existing evidence suggested that moderate-intensity aerobic exercise (60–75% of maximum heart rate), performed 3–5 times per week for 30–60 min per session, was especially beneficial for improving age-related SWS reduction in middle-aged and older adults (53). High-intensity exercise might be associated with reduced SWS due to increased sympathetic activation, particularly when performed in the evening (23). Resistance exercise showed modest associations with improved sleep efficiency but no significant associations with SWS (54). These findings suggested a non-linear relationship between exercise dosage and sleep architecture, and moderate-intensity exercise exerted the most favorable effects on SWS optimization (55).

### 3.2 Circadian timing of exercise

The cognitive associations of exercise might depend on its alignment with circadian rhythms (27). Morning exercise accompanied by light exposure could advance circadian phase, improve sleep onset, and extend SWS duration—making it potentially suitable for older adults with an evening chronotype (56). Evening exercise might increase core body temperature, delay sleep onset, and shorten SWS, though it may increase REM sleep density, with unclear implications for cognitive flexibility (57). Studies on rest-activity rhythm (RAR) supported this view: older adults with earlier peak activity time, higher activity amplitude, and better rhythm stability showed better overall cognitive performance (58), indicating that exercise regularity was equally important as total exercise volume for sleep and cognitive improvement (59).

### 3.3 Matching interventions to sleep disorder types

**Figure 2:**
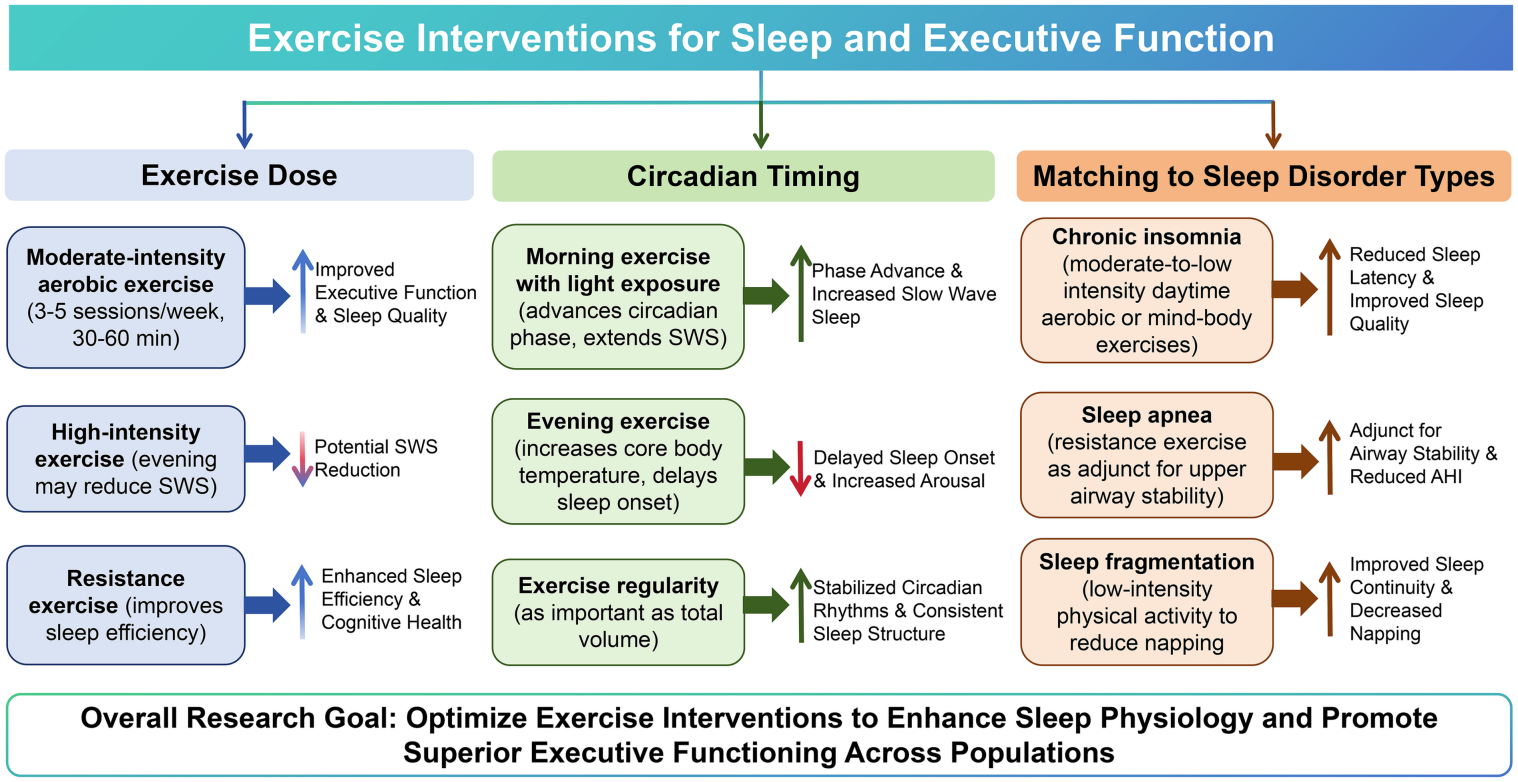
Dose, timing, and type matching of exercise interventions for sleep and executive function. The figure summarizes three key factors for exercise interventions targeting sleep and executive function: moderate-intensity aerobic exercise benefits slow-wave sleep, morning exercise suits older adults with an evening chronotype, and different sleep disorder types should be matched with appropriate exercise modalities (e.g., daytime aerobic exercise for insomnia, resistance training as an adjunct for sleep apnea, low-intensity activity for sleep fragmentation).

Chronic insomnia was characterized by anxious arousal and hypothalamic–pituitary–adrenal axis hyperactivation (60). Moderate-to-low intensity aerobic exercise—particularly when performed during the day—might improve sleep by reducing anxiety and regulating cortisol rhythms (61). Mind–body exercises such as Tai Chi and Qigong also showed clear associations with improved insomnia (62). Sleep apnea involved intermittent hypoxia and sleep fragmentation. Resistance exercise might partially alleviate symptoms by improving upper airway stability (63), with postural training and weight loss as core interventions, and exercise serving as an adjunct (64). Sleep fragmentation—common in older adults—is characterized by frequent nocturnal awakenings. Low-intensity physical activity might reduce daytime napping and increase nocturnal sleep pressure, thereby improving sleep continuity (65). Figure 2 illustrated the three key considerations for exercise interventions targeting sleep architecture and executive function: optimal dose, circadian timing, and matching to specific sleep disorder types.

## 4 Future research directions

Future studies should prioritize several key research priorities. First, well-designed randomized controlled trials combining exercise intervention with sleep manipulation (e.g., sleep extension, selective sleep deprivation and pharmacological sleep regulation) were required to verify whether exercise-induced cognitive improvements were mediated by sleep architecture changes, while accounting for potential bidirectional and reciprocal interactions (10). Second, longitudinal repeated-measures designs with multi-timepoint assessments of sleep architecture, physical activity and executive function helped clarify temporal causality and distinguish unidirectional, bidirectional and reciprocal relationships (66). Third, closed-loop intervention strategies that adjusted real-time exercise prescriptions based on wearable device-monitored sleep parameters (e.g., switching to low-intensity mind–body exercise after poor sleep) optimized the synergistic effects of exercise and sleep regulation (67). Moreover, multimodal approaches integrating functional magnetic resonance imaging, electroencephalography and actigraphy, combined with computational modeling, facilitated the construction of individualized response prediction models for combined intervention (68). Finally, the development of feasible, population-tailored intervention protocols (e.g., morning light-exercise combined therapy, midday short napping and evening relaxation training) and their translational application in community settings remained critical for clinical implementation. Targeted stratification for high-risk groups, such as apolipoprotein E ε4 carriers and patients with hypertension and chronic insomnia, was also essential for verifying real-world intervention efficacy (69).

## 5 Limitations

Several limitations constrain the interpretability of this review. First, most cited studies are cross-sectional or observational, precluding causal inference and leaving unmeasured confounding unresolved. Second, the hypothesized pathway in which sleep architecture is involved in exercise effects on executive function lacks direct testing; no randomized controlled trial has manipulated sleep architecture within an exercise intervention to examine whether altering sleep architecture changes the cognitive outcomes, which would be necessary to support a causal role. Third, mechanistic evidence, particularly for the glymphatic system and lactate signaling, derives primarily from animal models, limiting direct translation to aging humans due to species differences. Fourth, individual differences (e.g., genetic background, cognitive reserve, comorbidities) in middle-aged and older adults and considerable heterogeneity in the operationalization of sleep architecture and executive function across studies further complicate synthesis.

## 6 Conclusion

This review summarized current evidence focusing on middle-aged and older adults, and elaborated the reciprocal interactions among sleep architecture, physical activity and executive function. This review discussed several potential mechanisms that may interact, including shared neuromodulatory substrates, sleep-dependent glymphatic clearance, and antagonistic regulatory effects of sleep deprivation and exercise on the dynamic balance of the CEN, DMN and SN. Subcomponent-specific associations and non-linear dose-temporal effects further refined this interactive framework. However, most existing evidence remained correlational, and the bidirectional nature of sleep-exercise interactions left causal directions undetermined. Future research should adopt longitudinal and interventional designs to clarify reciprocal causal pathways. Such efforts would promote the transformation of the current conceptual framework into empirically validated understandings of how physical activity and sleep architecture jointly modulate executive function during human aging.
